# Social support from different sources and its relationship with stress in spaceflight analog environments

**DOI:** 10.3389/fpsyg.2024.1350630

**Published:** 2025-01-16

**Authors:** Suzanne T. Bell, Steven R. Anderson, Peter G. Roma, Lauren Blackwell Landon, Sheena I. Dev

**Affiliations:** ^1^National Aeronautics and Space Administration (NASA), Behavioral Health and Performance Laboratory, Biomedical Research and Environmental Sciences Division, Human Health and Performance Directorate, NASA Johnson Space Center, Houston, TX, United States; ^2^KBR, Behavioral Health and Performance Laboratory, Biomedical Research and Environmental Sciences Division, Human Health and Performance Directorate, NASA Johnson Space Center, Houston, TX, United States

**Keywords:** social support, astronaut, stress, marriage and family, team dynamics

## Abstract

While there is a large body of research on social support in traditional work settings, less is known about how the unique context of long-term isolation and confinement affects perceived social support. The purpose of our research was to examine how perceptions of social support change over time, how they differ by source (i.e., public, organization, family/friends/colleagues, and other crewmembers), and the relationship between social support and stress. We collected data from 64 crewmembers in five spaceflight analog campaigns with restricted communication with outside sources. Results suggested that perceived social support declined over time for all sources, with declines more pronounced for external sources (public, organization, family/friends/colleagues) than for perceived support from other crewmembers. While perceived overall social support was unrelated to stress over time, social support from crewmembers was related to the stress levels reported by crewmembers in the evening. Our results are important as they: (a) empirically document the decline in perceived social support over time in extended isolation; (b) provide evidence for the critical importance of the role of fellow crewmembers in being able to provide social support in conditions of extended isolation and communication delay; and (c) underscore the need to more fully understand the dynamics between the crew and external sources of social support (public, organization, family/friends/colleagues) as well as how those relationships may be best supported for individuals who live and work in long-term isolation and confinement.

## 1 Introduction

Social support is an important aspect of positive work environments and is related to desirable work and non-work outcomes including well-being (Terry et al., 1993; Panaccio and Vandenberghe, 2009), job burnout (Etzion, 1984), job satisfaction (Baruch-Feldman et al., 2002), and stress (Viswesvaran et al., 1999). Social support refers to the psychological or material resources provided to an individual by partners in a social relationship (Jolly et al., 2021). Individuals can draw on others for emotional support such as empathy, love, caring, and trust as well as instrumental forms of support such as time or other resources (e.g., how to approach a challenge). This support helps individuals meet basic needs of competence, autonomy, and relatedness that enable effective functioning (Basic Needs Theory; Ryan and Deci, 2000). In the workplace, social support can also help individuals meet job demands. For example, the Job Demands-Resources model and related research describes how social support and other job resources combine with job demands to predict outcomes at work including engagement, burnout, and job performance (Bakker and Demerouti, 2007).

While social support is important in general, its importance can be heightened for individuals who work in isolated and extreme environments because of the increased stressors as well as restricted communication with different sources of social support. Social support is particularly important in high stress environments. As examples, social support is a buffer of the stress and strain relationship in Army units (Bliese and Britt, 2001). Social support from a supervisor can reduce symptoms of post-traumatic stress disorder (PTSD) in emergency personnel (Ogińska-Bulik, 2013). Social support is also the primary coping mechanism astronauts report using in response to stressors in space (Suedfeld et al., 2009).

Social support can be provided by different sources including coworkers, organizational representative such as supervisors, and family and friends. Different sources of support tend to be moderately correlated but distinct (Abbey et al., 1985; Skomorovsky, 2014) and influenced by individual factors including personality (Caldwell and Reinhart, 1988). Meta-analyses have shown that the relationship between social support and outcomes varies as a function of the source (e.g., supervisor, coworker, organization; French et al., 2018). For example, in the high stress/high consequence environment of medical training, individuals indicated peers and mentors were a preferred source of social support for workplace issues (Mikkola et al., 2018). In military units, group leaders and the unit can both be important sources of social support (Bliese and Britt, 2001; Britt et al., 2004; Zang et al., 2017). In spaceflight, families and friends can be an important source of social support (Suedfeld et al., 2009; Deming and Vasterling, 2017), with the astronaut’s ability to call down to family and friends for a real-time conversation (Stuster, 2010, 2016). Eliciting social support from the general public through social media use may enhance the meaningfulness of astronauts’ work in space (Britt et al., 2017; Stuster, 2010, 2016).

While it is important to theoretically and practically distinguish sources of social support in general, understanding perceived social support across different sources may be even more important for those living and working in isolated settings within which communication with outside parties is limited because of a communication delay or restricted contact schedule. Aboard the International Space Station, crewmembers have real-time access to important sources of social support such as family and friends, the public (e.g., social media), and medical and psychosocial support services. Yet, for future space exploration missions in which a crew travels into deep space, the sheer distance from Earth will create a communication delay. For Moon missions one-way communication delays may be a few seconds; however, communication delays are estimated to be up to 22 min. each way for missions to Mars. There may also be substantial blackout periods when there is no communication with Earth. Real-time access to sources of social support including mission control, medical and psychological support services, and family and friends will be impossible on deep space missions. The dynamics of social support from different sources in such isolated and extreme circumstances is unknown.

Beyond significant communication constraints, crewmembers of future space exploration will face unprecedented challenges including high and variable workloads, living and working in space with other crewmembers for an extended period, and executing complex tasks more autonomously. As NASA returns to the Moon, crewmembers will live and work in vehicles and habitats significantly smaller than the International Space Station. The workload is anticipated to be high, with high frequency spacewalks to explore the Moon’s surface and build the infrastructure to create a sustained human presence on the Moon. For future space exploration missions such as a mission to Mars, the crew will live and work together for up to 2.5 years in a small space. Extended confinement in which crewmembers both live and work together will require that crewmembers be effective teammates and roommates (Landon et al., 2023). Whereas in a more traditional work setting, individuals may go home at the end of a workday and rely on family and friends to cope with workplace frustrations, they may need to self-regulate or rely on other crewmembers for emotion-focused social support when contact with Earth and important sources of social support is no longer available in real time. Problems back home may also be a source of stress for individuals in extreme environments and individuals may rely on other crewmembers for social support if the support provided by family and friends diminishes. Relying on other crewmembers for social support may be particularly helpful given how challenging it may be for those outside the extreme environment to understand the complexity of working in the operational environment. Further, external social support can be in real time and easily accessible, whereas communication with external sources can be structured in terms of when it is provided and limited to virtual modes of communication. Although there is evidence that even virtual sources of social support can buffer stress (Kothgassner et al., 2019), less is understood about prolonged reliance on virtual social support for individuals living in isolated and confined spaceflight-like conditions. Even seemingly simple tasks can become more complicated in a microgravity environment. This could create a situation in which other sources of organizational support such as management may be perceived to be able to provide less instrumental support. As an example, veteran astronauts often orient newer astronauts to the ‘tricks’ of effectively and more efficiently using the restroom in microgravity when they first arrive in space. For Mars mission, the crew will have to operate with unprecedented levels of autonomy and execute complex feats such as landing on Mars and spacewalks with no real-time support from Mission Control Center (MCC).

Taken together, the conditions expected for future space exploration creates a situation where a stressful work environment may have additional needs and demands that require additional social support to cope, yet there will be limited access to different sources of social support. Higher unmet needs (e.g., need for relatedness), more intensive environmental and job demands, and less availability of different sources of social support have the potential to result in lower perceived social support over time in isolated and confined settings. Thus, we investigate:

*H1:* Perception of social support will decline over time in extended isolation and confinement.

*RQ1*: To what extent do perceptions of social support change over time by source?

While it is important to characterize the changes in perceived social support over time from different sources for individuals in long-term isolation and confinement, it is also important to understand to what extent these changes are related to stress. Stress is a primary outcome of interest in social support research. Results of a large-scale meta-analysis suggest that social support can reduce the strain experienced, mitigate perceived stressors, and moderate the stressor-strain relationship (Viswesvaran et al., 1999). As the perceived availability of social support declines, there may be less available resources to meet the demands of the job and environment. Thus, we examine the relationship between perceptions of social support and stress over time and hypothesize:

*H2:* Perceived social support will be negatively related to stress over time in isolated and confined environments.

*RQ2:* To what extent does the perceived social support and stress relationship change as a function of source?

## 2 Method

### 2.1 Participants

Participants were 64 analog crewmembers in five spaceflight analog campaigns: NASA Human Exploration Research Analog (HERA) Campaigns 4, 5, and 6, and NASA/IBMP Scientific International Research In a Unique terrestrial Station (SIRIUS) Campaigns 19 and 21. Participants were 39.1% female, with an average age of 37.2 years (SD = 6.87). Forty-seven percent of participants identified as single or never married. A total of 67.2% of participants reported that they were currently in a committed romantic relationship. Crewmembers had an average of 0.69 months (SD = 3.29) of previous experience living in spaceflight analog environments and an average of 1.91 months (SD = 3.31) of previous experience living in confined or isolated operational environments (e.g., submarine, polar station, aircraft carrier, deep sea diving chamber, forward operating base, oil rig).

### 2.2 Spaceflight analog environments

Participants in HERA campaigns (*N* = 52) were confined to the HERA habitat at NASA Johnson Space Center for 45 days. Data were collected across three Campaigns, each with a primary manipulation described next and each with four missions (except for HERA Campaign 4, which had 5 missions). For all missions, contact with the outside world was controlled and limited. HERA crews were able to interact regularly with MCC and had once a week Private Psychological Conferences (PPCs), Private Medical Conferences (PMCs), and Private Family Conferences (PFCs). PFCs were conducted in a private location in the habitat (airlock) and were limited to audio and text communication. No other communication with family or friends was nominally allowed outside of PFCs.

HERA Campaign 4 (2017–2018) had a significant sleep deprivation manipulation in which crewmembers were limited to 5 h of sleep Monday through Friday and allowed 8 h on Saturday and Sunday. A communication delay was phased in from Mission Day (MD)2—MD19, peaked at 5 min. one-way from MD20—MD24, and phased out from MD25—MD44. Campaign 5 (2019–2020) had a privacy manipulation in which the environment was modified to have less privacy when using the restroom and sleeping. A communication delay was phased in from MD16—MD19, peaked at 5 min. from MD20—MD25, and phased out from MD26—MD29. Campaign 6 (2021–2023) included an autonomy manipulation in which crews were expected to work more autonomously from MCC than in other times in the mission. A communication delay was phased in from MD8—MD18, peaked at 5 min. from MD19—MD27, and phased out from MD28—MD38. In HERA C4 and C5, the communication delay was not applied to PFC audio loops; thus all PFCs were conducted in real time. In HERA C6, the communication delay active at that mission day was applied to PFC audio loops.

SIRIUS is a spaceflight analog in the Nazemnyy Eksperimental’nyy Kompleks (NEK) facility in Moscow, Russia. SIRIUS19 was a four-month (120 day) mission with a six-person international crew held in 2019. For SIRIUS19, a 5 min. one-way communication delay was implemented from MD11—MD108. SIRIUS21 was an eight-month (240 day) mission with a six-person international crew held from 2021 to 2022. In SIRIUS21, a 5 min. communication delay each way was implemented from MD8—MD236.

All analog campaigns were designed to simulate the stressful living and working conditions that astronauts would face in real spaceflight environments. Toward this goal, crewmembers were given spaceflight realistic workloads and occasionally encountered unannounced stressor events requiring timely responses and operational performance.

### 2.3 Measures

Data were collected as part of a larger Human Factors and Behavioral Performance Exploration Measures (HFBP-EM) protocol. HFBP-EM is a standard suite of measures collected in spaceflight analog environments. HFBP-EM assesses key metrics that characterize the Behavioral Health, Team, Sleep, and Human System Integration Architecture (HSIA) risks associated with spaceflight and enables the testing of countermeasures.

HFBP-EM surveys were examined to identify any response patterns that could suggest unmotivated or random participant responding. Response times and intra-individual response variability were assessed to identify potential careless responding (Dunn et al., 2018; Marjanovic et al., 2015). Observations identified as potentially careless were flagged and individually examined. A total of 10 observations were identified as careless using this methodology and were excluded from all analyses and figures in the present study.

### 2.4 Social support

Social support was operationalized as scores on a modified version of the ENRICHD Social Support Inventory (ESSI). The ESSI is a 7-item self-report survey that assesses perceived social support across multiple dimensions and sources (Mitchell et al., 2003). Crewmembers rated how much social support they received over six dimensions (listen, advice, love, chores, emotional, and contact). The ESSI was modified for use in spaceflight analog settings by removing the dichotomous item assessing marital status (since it was asked as part of a demographic questionnaire) and the addition of having crewmembers rate each item for each of four sources of social support (public, analog organization, family/friends/colleagues, and crew). The administration of the ESSI in HERA Campaign 4 did not include ratings for separate sources of social support. The 5-point original scale was modified to a 7-point scale with anchors at 1 = None of the Time, 4 = Some of the Time, and 7 = All of the Time. The scores of the 6 items were summed to create a total score ranging from 6 to 42 for each source, with higher scores indicating greater perceived social support. Social support was collected every 10 days in-mission during HERA campaigns, every 14 days during pre- and in-mission phases in SIRIUS19, and every 20 days during pre-, in-, and post-mission phases in SIRIUS21. We first examined social support (ESSI sum) averaged across all sources. We then examined social support separated by source.

### 2.5 Stress

We operationalized stress as a single item in which crewmembers were asked to rate their perceived stress on a 100-point Visual Analog Scale ranging from Not Stressed At All (0) to Very Stressed (100) (Basner et al., 2014). Stress was assessed daily in the morning and evening in HERA C4, C5, C6 and SIRIUS19, and every third day in the morning and evening in SIRIUS21.

### 2.6 Analytical approach

To examine our hypotheses and research questions, we used a series of descriptive figures as well as linear mixed model (LMM) analyses. In our models, we included crewmember-specific intercepts as a random effect because of anticipated individual differences in perceived social support. Mission day was included as a fixed effect to examine temporal trends. Campaign was included as a fixed effect to statistically control for any differences between campaigns. Communication delay in minutes was also included as a fixed effect to statistically control for the effect of communication delay on perceived social support. Multicollinearity among independent variables was assessed by calculating generalized variance inflation factors (VIFs) for each LMM predictor (Fox and Monette, 1992). Generalized GVIF1/(2·df) values <5 were interpreted as indicating no problematic multicollinearity. For each analysis, we plotted the residuals to check for non-normality. We used robust LMM when there were extreme datapoints and violations of normality (Koller, 2016). Statistical significance was set to *α* = 0.05. All analyses were performed using R 4.2.1 (R Core Team, 2022).

## 3 Results

Descriptive statistics for the primary outcome of perceived social support are presented in Table 1.

**Table 1:** Means, medians, and standard deviations for social support by source.

	Public (*N* = 558)	Organization (analog management) (N = 558)	Family/friends/colleagues (*N* = 558)	Crew (*N* = 558)	Overall (*N* = 3,173)
**Social support**					
Mean (SD)	17.9 (11.3)	27.3 (9.31)	33.4 (9.34)	34.7 (6.94)	28.5 (11.3)
Median [Min, Max]	15.0 [6.00, 42.0]	29.0 [7.00, 42.0]	36.0 [6.00, 42.0]	36.0 [12.0, 42.0]	31.0 [6.00, 42.0]

*N, number of observations.*

### 3.1 Perceptions of social support over time

**Figure 1:**
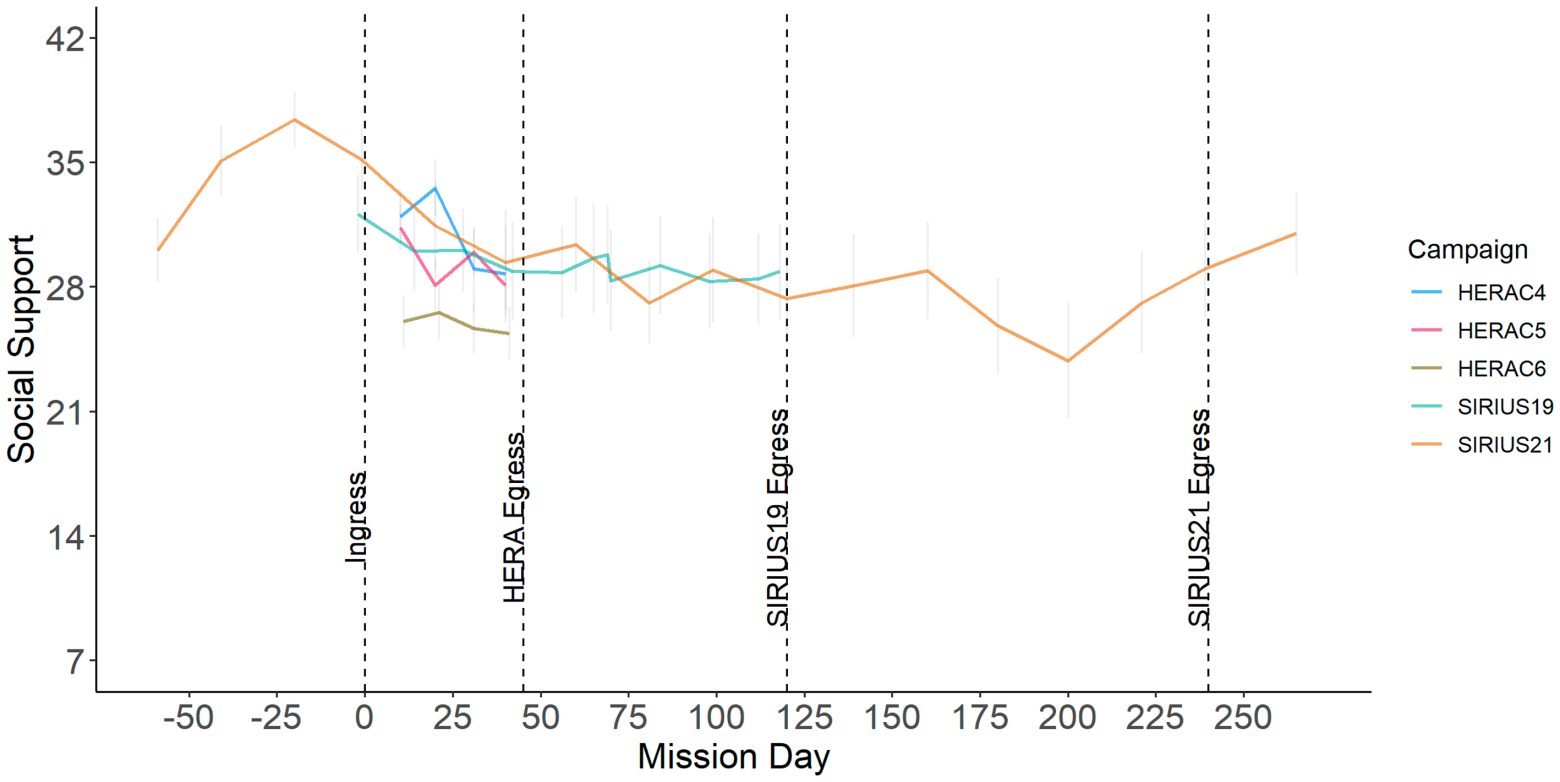
Social support averaged across all sources over mission.

Hypothesis 1 predicted that overall social support would decline over time in isolated and confined environments. Figure 1 shows a plot of perceived social support averaged across all sources (public, analog organization, family/friends/colleagues, and other crewmembers) by mission day, and suggests that overall perceived social support declined over time. Social support peaked around ingress and gradually declined over the course of the mission. In SIRIUS21, which assessed social support during pre-mission and post-mission phases, perceived social support increased after egress post-mission and gradually returned to pre-mission baseline.

To test Hypothesis 1, we conducted a robust linear mixed model (LMM) analysis in which we examined the relationship between time and perceptions of overall social support. Results are presented in Table 2 and, in support of Hypothesis 1, indicate that social support declined over time, *β* = −0.02, CI = −0.03 – −0.01, *t* = −3.77, *p* < 0.001.

**Table 2:** Results of robust linear mixed model (LMM) testing effect of time on perceived social support, controlling for campaign and communication delay.

Social support					
Predictors	Estimates	Std. Error	CI	Statistic	*p*
(Intercept)	32.0984	1.9765	28.2245–35.9723	16.2397	**<0.001**
Mission day	−0.0189	0.0050	−0.0287 – −0.0090	−3.7675	**<0.001**
Campaign [HERAC5]	−1.6798	2.7198	−7.0105 – 3.6509	−0.6176	0.537
Campaign [HERAC6]	−4.8106	2.7196	−10.1410 – 0.5198	−1.7688	0.077
Campaign [SIRIUS19]	−0.1994	3.5921	−7.2398 – 6.8409	−0.0555	0.956
Campaign [SIRIUS21]	1.4218	3.5737	−5.5824 – 8.4261	0.3979	0.691
Communication delay	−0.1718	0.1239	−0.4147 – 0.0712	−1.3857	0.166
**Random effects**					
*σ* ^2^	81.38				
τ_00 ID_	48.57				
ICC	0.37				
N _ID_	64				
Observations	1,165				
Marginal *R*^2^ / Conditional *R*^2^	0.034 / 0.395				

ID, subject. ICC, intraclass correlation coefficient. CI, confidence interval. HERA Campaign 4 acts as the intercept (reference). Bold values indicate statistical significance (*p* < 0.05).

### 3.2 Perceptions of social support by source over time

**Figure 2:**
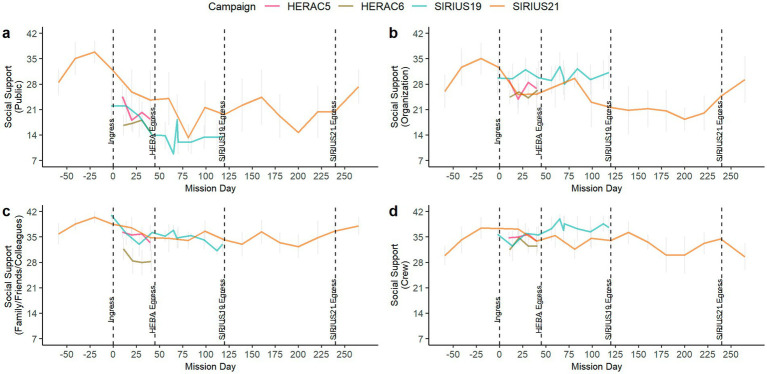
Social support by source over mission day; **(a)** Social support from the public over mission day; **(b)** Social support from the analog organization over mission day; **(c)** Social support from family over mission day; **(d)** Social support from crew over mission day.

Our next research question asked to what extent perceptions of social support changed over time by source. To examine this, we plotted perceptions of social support by campaign across mission day for each source of social support (i.e., public, analog organization, family/friends/colleagues, crew). Results are shown in Figures 2A–D and suggest that the change in perceived social support over time differed by the source of social support. Social support from the general public (e.g., social media, public outreach) steeply declined after ingress and only occasionally increased across campaigns over time, possibly in response to public outreach events (Figure 2A). Social support from the analog organization showed a similar pattern over time, generally decreasing after ingress but increasing at various points throughout the mission, possibly in response to contact with MCC. For example, an increase in perceived social support from the analog organization in the long-duration SIRIUS21 mission around MD80 may have been in response to MCC satisfactorily troubleshooting an issue with the psychological support computer server that the crew reported from MD74—MD76.

In contrast to support from the public or analog organization, social support from family (Figure 2C) and crew (Figure 2D) was more stable over the course of the mission across campaigns. In SIRIUS19, on average, social support from the family/friends/colleagues notably declined, while social support from crew increased over time. This may reflect a shifting of social support from external to internal sources as time spent living in isolation prolonged. Interestingly, this pattern was not as evident in the longer duration SIRIUS21 mission, suggesting that it may not always occur.

**Figure 3:**
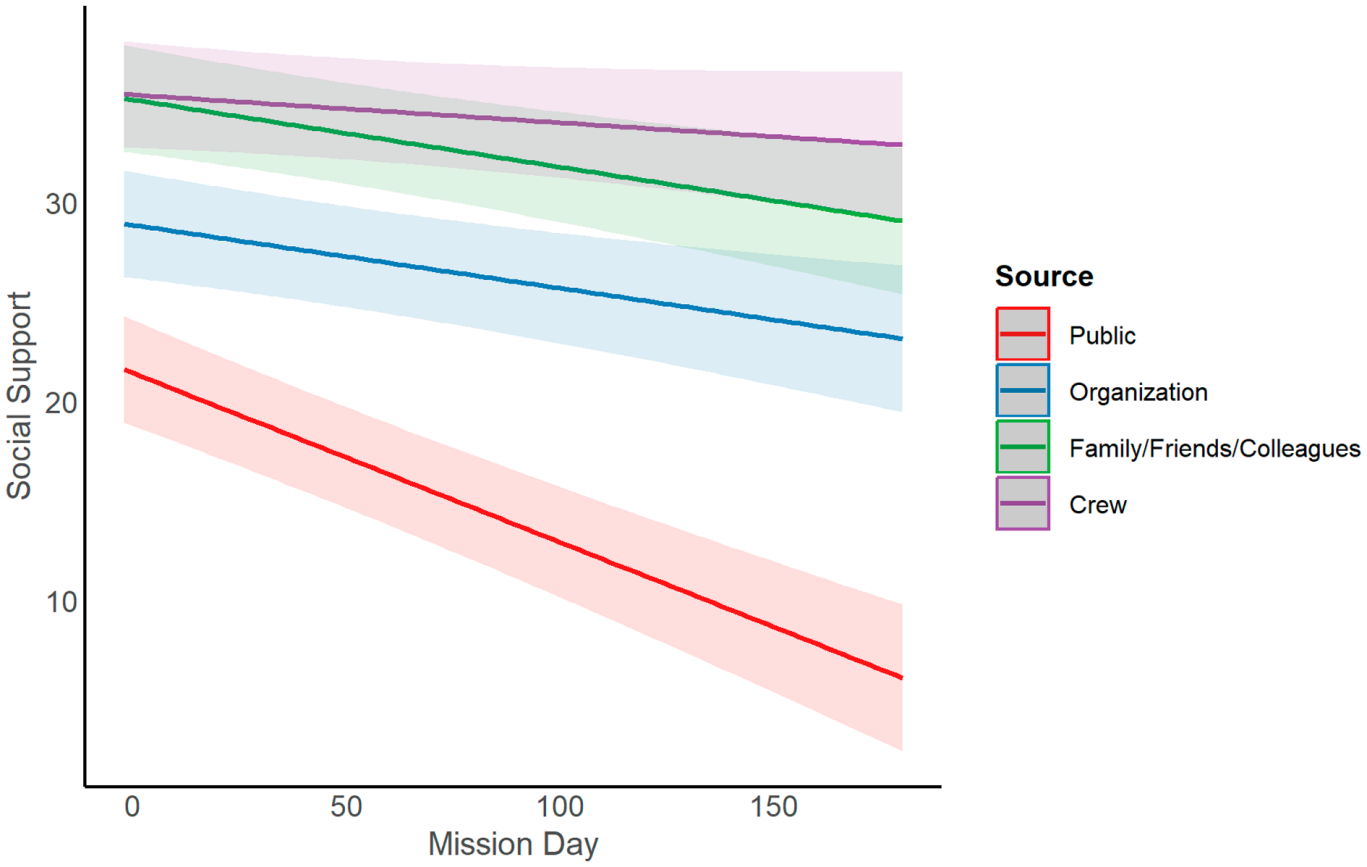
Interaction between time and source of social support predicting perceived social support.

We further examined Research Question 1 by specifying a robust LMM in which we examined the relationship between time and perception of each source of social support. We anticipated that there would be a significant decline in social support for outside sources compared to the crew, which we expected to vary more as a function of crew or individual. Results are presented in Table 3 and suggest that there was a significant decline in perceived social support over time for all sources. Plotting the interaction between time and source of social support (Figure 3) revealed that although all sources of social support declined over time, social support from the public showed the steepest decline over time, while social support from the crew was highest overall and declined slightly less over time compared to social support from family/friends/colleagues.

**Table 3:** Results of robust linear mixed model (LMM) testing interaction between time and source of social support on perceived social support, controlling for campaign and communication delay.

Social Support					
Predictors	Estimates	Std. Error	CI	Statistic	*p*
(Intercept)	21.6539	1.9850	17.7633–25.5445	10.9085	**<0.001**
Mission day	−0.0852	0.0099	−0.1046 – −0.0657	−8.5920	**<0.001**
Source [Org]	7.4348	0.7298	6.0043–8.8652	10.1868	**<0.001**
Source [Family/friends/colleagues]	13.7339	0.7284	12.3062–15.1616	18.8540	**<0.001**
Source [Crew]	13.9756	0.7306	12.5437–15.4076	19.1288	**<0.001**
Campaign [HERAC6]	−2.5402	2.7227	−7.8766 – 2.7962	−0.9330	0.351
Campaign [SIRIUS19]	1.9158	3.6798	−5.2964 – 9.1280	0.5206	0.603
Campaign [SIRIUS21]	3.9622	3.7180	−3.3250 – 11.2494	1.0657	0.287
Mission day × Source [Org]	0.0534	0.0129	0.0282–0.0786	4.1560	**<0.001**
Mission day × Source [Family/friends/colleagues]	0.0513	0.0128	0.0262–0.0765	3.9974	**<0.001**
Mission day × Source [Crew]	0.0712	0.0129	0.0460–0.0964	5.5385	**<0.001**
Communication delay	−0.1844	0.0809	−0.3429 – −0.0258	−2.2789	**0.023**
**Random effects**					
*σ* ^2^	45.04				
τ_00 ID_	54.93				
ICC	0.55				
N _ID_	44				
Observations	1,676				
Marginal R^2^ / Conditional R^2^	0.334 / 0.700				

ID, subject. ICC, intraclass correlation coefficient. CI, confidence interval. HERA Campaign 4 acts as the intercept (reference). Bold values indicate statistical significance (*p* < 0.05).

### 3.3 Relationship between perceived social support and stress

**Figure 4:**
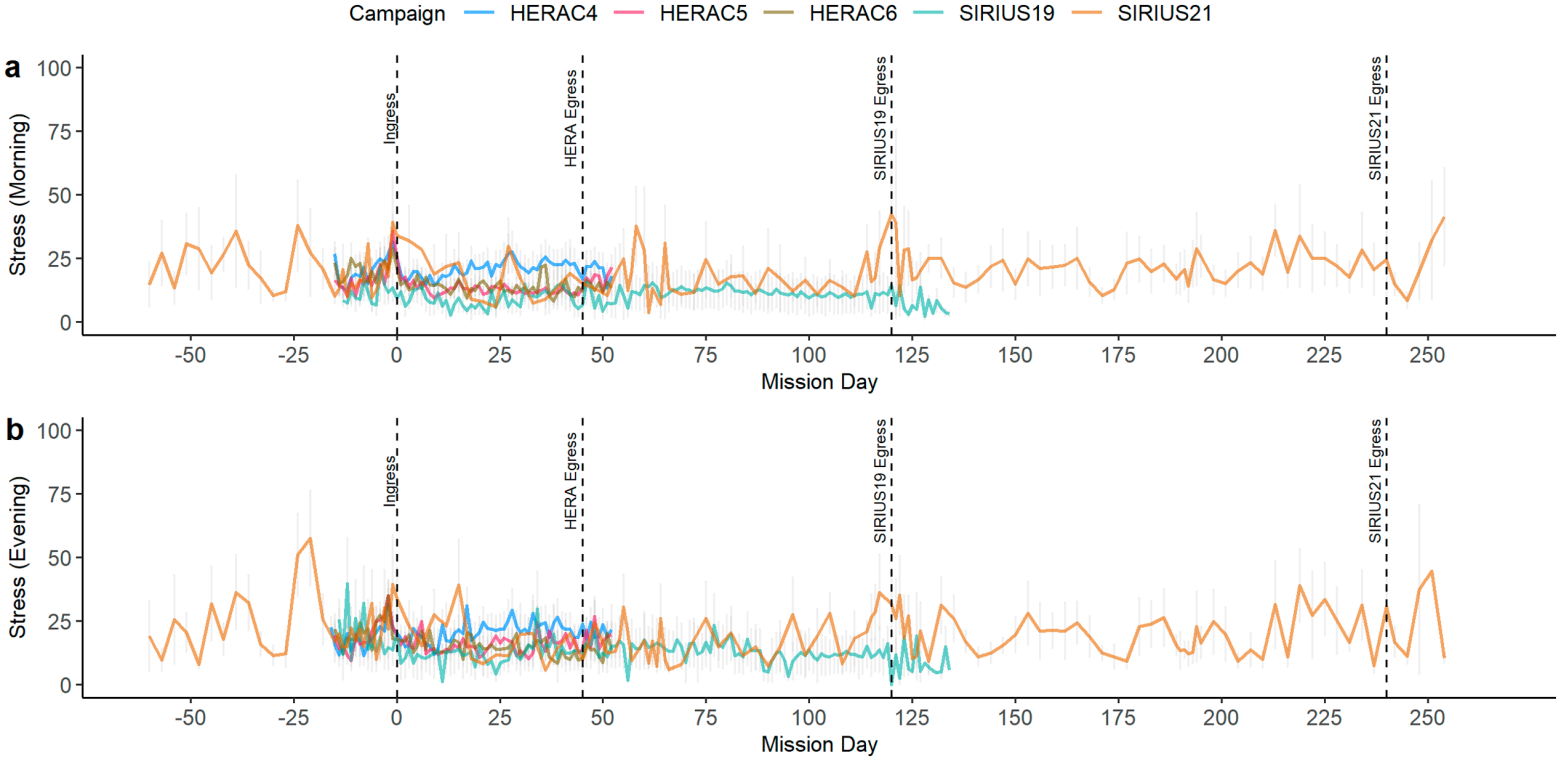
Stress by administration time over mission day; **(a)** Stress in the morning over mission day; **(b)** Stress in the evening over mission day.

Hypothesis 2 predicted that perceived social support would be negatively related to stress over time in isolated and confined environments. We examined Hypothesis 2 in stress data collected in the morning and in the evening in separate analyses. We first plotted stress reported in the morning and evening over time in all campaigns (Figure 4). There was considerable variability in both morning (Figure 4A) and evening (Figure 4B) reported stress, with stress levels highest in HERA C4, which had a notable sleep deprivation manipulation, and the SIRIUS 21 mission, which had the longest (240-day) isolation period in our dataset and an unexpected crewmember early egress.

To test the relationship between social support and stress (Hypothesis 2), we specified robust LMMs in which we included campaign and communication delay as controls and overall social support and mission day as predictors of stress. Stress reported in the morning significantly decreased over time, *β* = −0.02, CI = −0.03 – −0.02, *t* = −5.34, *p* < 0.001. Stress in the evening also significantly decreased over time, *β* = −0.02, CI = −0.03 – −0.01, *t* = −3.74, *p* < 0.001. However, perceived overall social support across all sources was not related to morning or evening stress.

Finally, to examine Research Question 2, which asked to what extent the perceived social support and stress relationship changed as a function of source, we specified robust LMMs with social support from the crew and mission day predicting stress, controlling for campaign and communication delay. We chose to focus on social support from the crew as it was the highest source of social support for crewmembers (Table 1) and represents the most proximal “internal” source of support for crewmembers in an isolated and confined environment. Thus, because of its central role in coping, we predicted that perceived social support from fellow crewmembers over the course of the mission would be related to stress. Consistent with this, results indicated that increased perceived social support from the crew was significantly related to less stress in the evening, *β* = −0.42, CI = −0.58 – −0.26, *t* = −5.11, *p* < 0.001 (Table 4). Social support from the crew was not related to stress reported in the morning, β = 0.01, CI = −0.15 – 0.17, *t* = 0.13, *p =* 0.895.

**Table 4:** Results of robust linear mixed model (LMM) testing relationship between social support from crew and stress reported in the evening, controlling for campaign and communication delay.

	Stress (Evening)				
Predictors	Estimates	std. Error	CI	Statistic	*p*
(Intercept)	24.98	4.15	16.84–33.12	6.02	**<0.001**
Social support [Crew source]	−0.42	0.08	−0.58 – −0.26	−5.11	**<0.001**
Mission day	−0.02	0.01	−0.03 – 0.00	−1.51	0.131
Campaign [HERAC6]	0.10	4.26	−8.26 – 8.45	0.02	0.982
Campaign [SIRIUS19]	1.49	5.76	−9.80 – 12.78	0.26	0.796
Campaign [SIRIUS21]	8.08	5.82	−3.33 – 19.48	1.39	0.165
Communication delay	0.08	0.14	−0.19 – 0.35	0.59	0.554
**Random effects**					
*σ* ^2^	16.05				
τ_00 ID_	133.89				
ICC	0.89				
N _ID_	44				
Observations	207				
Marginal R^2^ / Conditional R^2^	0.084 / 0.902				

ID, subject. ICC, intraclass correlation coefficient. CI, confidence interval. HERA Campaign 4 acts as the intercept (reference). Bold values indicate statistical significance (*p* < 0.05).

## 4 Discussion

The objective of our research was to examine how perceptions of social support change over time, how they differ by source (i.e., public, analog organization, family/friends/colleagues, and other crewmembers), and the relationship between social support and stress for crewmembers in isolated and confined environments. We examined these relationships using data from crewmembers confined to small, communication-delayed, simulated space habitats with restricted contact to sources of social support outside the habitat and mission lengths of up to 240 days. We first found that perceived social support, averaged across all sources, declined over time in all spaceflight analog campaigns. In a follow-up analysis, we found a significant interaction between time and source of social support predicting perceived social support. Although all sources of social support declined over time, social support from the public showed the steepest decline over time, while social support from the crew was highest overall and declined slightly less over time compared to other sources. However, when we examined the relationship between perceived social support across all sources and stress, no relationship was observed. When examining the relationship between perceived social support and stress by source, we found that increased perceived social support from fellow crewmembers was related to less stress reported in the evening.

Our research makes several contributions. First, we were able to empirically document the decline in perceived social support over time in isolation. Research to date in isolated and confined environments, such as McMurdo Station, Amundsen-Scott South Pole Station, and Belgrano II Argentine Station in Antarctica, has shown decreased social support (Tortello et al., 2021) and decreased satisfaction with social support over time (Palinkas et al., 2004). In our research we were able to examine perceptions of social support over time in multiple isolated and confined spaceflight analog environments with individuals in isolation up to 240 days. The decline in social support over time underscores the importance of examining these issues in long-duration analogs.

Second, we provide evidence for the critical role that fellow crewmembers may provide in extended isolation. Low team cohesion or the emergence of isolates within a team may erode a critical source of social support for individuals in long-term isolation. Prior research has shown that crewmembers struggling with mood or interpersonal issues related to prolonged isolation may not make effective sources of social support (Palinkas et al., 2004). Increased workload and stress over time may also lead to conservation of resources and result in less frequent or meaningful social interactions over time (Westman et al., 2004). Despite this, social support from other crewmembers is an important contributor to crew behavioral health during missions (Tortello et al., 2021). In our research, declines were observed for some crews in long-duration isolation but not others, despite similar mission profiles. One potential explanation is that team dynamics moderated a shift from external to internal sources of social support over time. Additionally, individuals living and working in isolated and confined environments may rely more on their fellow crewmembers to compensate for perceived reductions in social support from other sources not in the extreme environment. Support from fellow crewmembers may also be more significant for reducing stress due to its accessibility and proximity compared to external sources of support. The shift from external to internal sources of social support underscores the importance of selection, training and other intervention of crew that support positive crew dynamics, and consideration of whether crewmembers are viewed as trusted partners and possible sources of social support in crew assignment.

Third, there was a significant decline, on average, for other sources of social support including the public, family and friends, and organizational management. While crewmembers may provide a significant and important source of social support for fellow crewmembers, it may still be important to ensure perceptions of other sources of social support are able to thrive in missions with extended isolation. For example, declines in social support from the organization and management may be problematic if it means that crewmembers do not believe that mission control will be able to provide instrumental support such as information appropriate for problem solving. Psychological closing, or the decreased communication through isolation, decreased number of issues discussed, and showing preference for specific communication partners (Gushin et al., 2012), may be related to less perceived social support over time. The decreased communication volume by commanders in their written reports have been observed in written reports to mission control but not audio logs (Bell et al., 2019). For future exploration missions, additional research is needed to understand how different forms of communication may help relationships thrive between the crew and Earth, particularly under conditions of communication delay. Training individuals who provide external sources of support such as mission control in perspective taking and other skills could improve perceived social support. Declines in perceived social support from family and friends may suggest a disconnect in shared identity and closeness that could become more pronounced on extended missions such as a mission to Mars. Future research is needed to understand how to best support these different relationships (e.g., pre-mission intervention, communication approaches) under the conditions expected for future space exploration (e.g., isolation, communication delay).

There are a few limitations worth noting that can also lead to potentially fruitful areas of future research. First, our use of a single item global measure of stress limits our ability to differentiate between different types and causes of stress. Second, all data in the present analyses were from astronaut-like individuals living in isolated and confined spaceflight simulated habitats. Several factors relevant to perceived social support and stress may differ between spaceflight analog participants and astronauts in space. These may include the frequency and significance of public outreach events affecting perceived social support from the public and the higher stress and real-life consequences of living and working in microgravity compared to a spaceflight analog. Thus, while our research provides an important step in characterizing how perceptions of social support across different sources change over time in extended isolation, additional work is needed with the astronaut population. Finally, our finding that social support from crewmembers was associated with stress in the evening, but not the morning, may be due in part to the closer temporal proximity of when the social support survey was administered (i.e., in the afternoon or evening).

In our study we focused on perceptions of social support. There has been some work distinguishing between actual and perceived social support. In our study, we focused on perceptions, which is particularly important given that external sources may still wish and attempt to provide social support, but the individual may not perceive it to be available. Future research could examine the presence or absence of objective social support behaviors (e.g., by quantifying the amount, content, and depth of communication) and how its presence from different sources (crew, family and friends) relates to perceived social support and outcomes such as stress. A focus on social support behaviors could also help to identify crewmember behaviors that contribute to a positive work environment.

Finally, in our models we included a random effect to account for variability between individuals. In our linear mixed models, intraclass correlation coefficients (ICCs) representing the proportion of the variance explained by the grouping structure (participant ID) in the population ranged from 0.37 to 0.89, suggesting that there was a considerable amount of inter-individual variability. While we statistically accounted for this dependence in the data, future research can explore individual difference factors in perceived social support, how these may impact the drawing of social support across different sources, as well as how these may moderate the relationship between social support and stress.

Despite these limitations, we believe our research makes a substantial contribution in characterizing crewmembers’ perceptions of social support across different sources, and the dynamics of how these perceptions may change over time in extended isolation. Further, our data suggest that social support can help provide a positive work environment in remote, isolated, and extreme environments by minimizing negative aspects (i.e., reported stress) experienced in these environments so that positive benefits and mission accomplishment can be achieved.

## Data Availability

The datasets presented in this study can be found in online repositories. The names of the repository/repositories and accession number(s) can be found at: https://nlsp.nasa.gov/explore/lsdahome/datarequest.
